# Sex-Gender Differences Are Completely Neglected in Treatments for Neuropathic Pain

**DOI:** 10.3390/ph17070838

**Published:** 2024-06-26

**Authors:** Francesco Salis, Salvatore Sardo, Gabriele Finco, Gian Luigi Gessa, Flavia Franconi, Roberta Agabio

**Affiliations:** 1 Department of Biomedical Sciences, Section of Neurosciences and Clinical Pharmacology, University of Cagliari, 09042 Monserrato, CA, Italy; 2 Department of Medical Sciences and Public Health, University of Cagliari, 09042 Monserrato, CA, Italy; salvatore.sardo@unica.it (S.S.); gabriele.finco@unica.it (G.F.); 3 Neuroscience Institute, Section of Cagliari, National Research Council, 09042 Monserrato, CA, Italy; lgessa@unica.it; 4 Laboratory of Sex-Gender Medicine, National Institute of Biostructures and Biosystems, 07100 Sassari, SAR, Italy; franconi.flavia@gmail.com

**Keywords:** neuropathic pain, pharmacological treatment, sex-gender differences

## Abstract

As sex-gender differences have been described in the responses of patients to certain medications, we hypothesized that the responses to medications recommended for neuropathic pain may differ between men and women. We conducted a literature review to identify articles reporting potential sex-gender differences in the efficacy and safety of these medications. Only a limited number of studies investigated potential sex-gender differences. Our results show that women seem to achieve higher blood concentrations than men during treatment with amitriptyline, nortriptyline, duloxetine, venlafaxine, and pregabalin. Compared to men, higher rates of women develop side effects during treatment with gabapentin, lidocaine, and tramadol. Globally, the sex-gender differences would suggest initially administering smaller doses of these medications to women with neuropathic pain compared to those administered to men. However, most of these differences have been revealed by studies focused on the treatment of other diseases (e.g., depression). Studies focused on neuropathic pain have overlooked potential sex-gender differences in patient responses to medications. Despite the fact that up to 60% of patients with neuropathic pain fail to achieve an adequate response to medications, the potential role of sex-gender differences in the efficacy and safety of pharmacotherapy has not adequately been investigated. Targeted studies should be implemented to facilitate personalized treatments for neuropathic pain.

## 1. Introduction

Pain represents the main reason for seeking medical treatment, with more than 30% of people suffering from chronic pain, defined as a painful condition that persists beyond 3 months [1]. Chronic pain is usually classified into nociceptive (related to tissue injury, mainly due to degenerative osteoarticular diseases, trauma, and visceral pathology), nociplastic (related to a sensitized central nervous system, such as fibromyalgia and some tension-type headaches), and neuropathic pain (caused by injury or a disease of the somatosensory nervous system, including carpal tunnel syndrome, diabetes, and chronic post-surgical pain) [1,2,3]. However, more than 50% of pain conditions (especially those involving cancer and spinal pain) have a mixed pain phenotype [1]. As an example, chronic degenerative osteoarticular pain may be nociceptive, inflammatory, and/or neuropathic pain.

Neuropathic pain affects up to 10% of the general population, more frequently women, the elderly population, people with diabetes mellitus, and those undergoing chemotherapy, accounting for millions of people worldwide [4]. As an example, neuropathic pain occurs in approx. 90% of diabetic patients (one of the third-millennium pandemics) [5], more frequently among female patients rather than male patients [6,7,8]. Despite its huge prevalence worldwide and higher frequency among women, the molecular bases and reasons underlying sex-gender differences in neuropathic pain remain poorly understood [9,10,11]. In this manuscript, the term “sex-gender differences” is used as the term “sex” is confined to the biological body while “gender” includes socioeconomic status, income, education, neighborhood characteristics, lifestyles, and environmental exposures. Sex and gender interact to form gordian nodes. Therefore, several authors have proposed use of the term “sex-gender-based medicine” [12,13]. Here, the term “sex-gender differences” is used to include both biological and social-cultural-economic contexts.

Historically, males have been utilized as the predominant or unique sex in both preclinical and clinical studies, with the result being that the female presentations and responses to treatment of several diseases, including pain, are often completely unknown [14,15]. Recent findings report how the main sex-gender differences in neuropathic pain seemingly depend on microglia and inflammation [11,14]. Clinically, further to differences in prevalence [4], sex-gender differences have been observed in the pathophysiology and intensity of neuropathic pain [6,7].

Amongst chronic pain syndromes, neuropathic pain often constitutes a devastating experience for patients and causes them to be affected by a severe reduction in their quality of life, representing a clinical challenge for their healthcare providers, especially considering the sobering results of available treatments [16,17,18]. Indeed, up to 60% of patients with neuropathic pain fail to achieve an adequate response to the medications recommended to treat this pain condition [19]. As previously demonstrated for other medications [11,13,20,21], we hypothesized that responses to these medications may differ between men and women. To evaluate this hypothesis, we conducted a comprehensive literature review aimed at investigating sex-gender differences in the efficacy and safety of medications recommended for the treatment of neuropathic pain.

## 2. Relevant Sections

**Figure 1:**
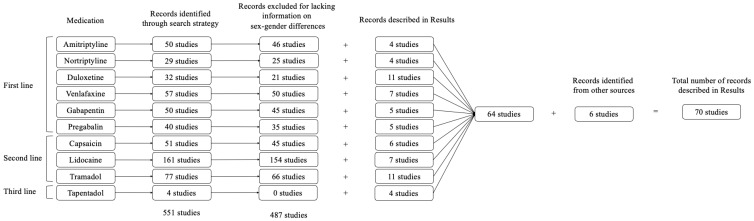
Search flow diagram.

For each medication, we first gathered evidence for its recommended use in the treatment of neuropathic pain based on recent systematic reviews on this topic, regardless of any potential sex-gender differences. Then, we conducted a search to identify potential sex-gender differences in the efficacy and safety of these medications. Data were obtained by searching the published medical literature in MEDLINE via PubMed from its inception up to 23 November 2023. Search terms included the following: the name of the individual medication (i.e., amitriptyline, nortriptyline, duloxetine, venlafaxine, gabapentin, pregabalin, tapentadol, tramadol, lidocaine, or capsaicin) AND (sex differences or gender differences), with the filters “Humans” and “English”. The best-quality evidence (e.g., meta-analyses, systematic reviews) received the greatest emphasis. We excluded reports and studies that did not provide information or did not investigate potential sex-gender differences (see Figure 1). Then, we illustrated the results of our search describing the sex-gender differences found. Data sheets of each medication were also evaluated to investigate recommendations for their use during pregnancy and breastfeeding.

## 3. Recommendation for the Treatment of Neuropathic Pain

One systematic review (SR) aimed at providing recommendations for the pharmacotherapy of neuropathic pain evaluated over 220 randomized controlled trials (RCTs) addressing the efficacy and safety of several medications [18]. Based on the results of this SR, amitriptyline, nortriptyline, duloxetine, venlafaxine, gabapentin, and pregabalin should be considered first-line options for the treatment of neuropathic pain (strong recommendations; Table 1), with topical capsaicin, topical lidocaine, and tramadol being second-line options (weak recommendations), and tapentadol being a third-line option (weak recommendation) [18]. This study also estimated the number needed to treat (NNT; the number of patients that need to be treated to obtain therapeutic effect in one patient; a lower NNT implies a higher efficacy) for each different medication and assigned the lowest value (3.6) to combined tricyclic antidepressants (amitriptyline and nortriptyline) and the highest value to topical capsaicin (10.6) (Table 1) [18]. However, other SRs report only partially overlapping findings.

Regarding first-line options, two Cochrane SRs focused on the efficacy of amitriptyline (17 RCTs; more than 1300 patients) [22] and nortriptyline (6 RCTs; more than 300 patients) [23] found little evidence to support their use in the treatment of neuropathic pain. Another Cochrane SR (43 RCTs; more than 6000 patients) concluded that duloxetine is the antidepressant with the highest certainty of evidence in the treatment of chronic pain [24], while another (6 RCTs; less than 500 patients) stated that evidence of the efficacy of venlafaxine in the treatment of neuropathic pain is lacking [25]. A different Cochrane SR (37 RCTs; almost 6000 patients) revealed that gabapentin can provide a good neuropathic pain response (3–4 patients out of 10 achieved at least a 50% reduction in pain), particularly in some phenotypes of postherpetic neuralgia and diabetic neuropathy [26]. Finally, another Cochrane SR (45 RCTs; almost 12,000 patients) confirmed the efficacy of pregabalin in several forms of neuropathic pain [27]. However, the NNT values for each typology of neuropathic pain ranged from 2.7 (in reducing pain intensity by at least 30% among patients with postherpetic neuralgia) to 22 (in reducing pain intensity by at least 30% among patients with painful diabetic neuropathy) [27].

Regarding second-line options, capsaicin, an active ingredient of chili peppers, exhibits agonist activity on Transient Receptor Potential (TRP) vanilloid receptor type 1 (TRPV1) [28]. The function of TRP channels can be differently modulated in men and women, contributing to sex differences in chronic pain and underlining the need to identify personalized chronic pain treatments [29]. One Cochrane SR (eight RCTs; 2500 participants, 36.6% women) showed a higher efficacy of high-concentration topical capsaicin compared to lower concentrations of capsaicin without investigating potential sex-gender differences [30]. Another recent SR (27 studies; 358 participants) reported that there is insufficient evidence to recommend lidocaine infusion in the treatment of chronic neuropathic pain [31]. Similarly, another Cochrane SR (six RCTs; 212 women and 226 men) assigned a low amount of evidence to the efficacy of tramadol in reducing neuropathic pain, mainly due to the limited number of participants and studies [32]. Finally, although tapentadol is recommended as a third-line option, one narrative review of RCTs highlighted its efficacy in treating neuropathic pain [33].

Despite these partially contrasting results, none of these SRs investigated the potential role of sex and gender in the efficacy and safety of medications recommended for the treatment of neuropathic pain [18,22,23,24,25,26,27,28,29,30,31,32,33].

## 4. Evidence of Sex-Gender Differences

### 4.1. Amitriptyline

#### 4.1.1. Studies Focused on the Treatment of Neuropathic Pain

We did not detect any study investigating or reporting evidence of sex-gender differences in the efficacy and safety of amitriptyline to treat neuropathic pain.

#### 4.1.2. Studies Focused on Diseases Other than Neuropathic Pain

One narrative review described potential sex-gender differences in the pharmacokinetics and pharmacodynamics of medications used to treat itching, including amitriptyline [34]. Using dose-corrected serum concentrations, women achieved higher levels of amitriptyline than men (see Table 1), as reported in a retrospective analysis of 396 women and 297 men taking amitriptyline plus nortriptyline [35]. Higher differences in amitriptyline levels were observed between older women (older than 60 years) and younger men (younger than 60 years).

Another clinical study investigated possible sex-gender differences in the association between the use of antidepressants, including amitriptyline, and restless legs syndrome [36]. Almost 1700 veterans (400 women; 1300 men) were interviewed and information from their medical records was collected. Only 6 women and 19 men from this large sample had received amitriptyline. The results showed how the use of amitriptyline was associated with restless legs syndrome in men but not in women. However, the scarce number of participants limits the validity of these results.

#### 4.1.3. Data Sheet

The amitriptyline data sheet includes no specific information regarding its use in the female population, except for pregnant and breastfeeding women (see Table 1). Specifically, amitriptyline should only be used in pregnancy if considered necessary, given the risks of untreated depression, and under close medical supervision. Regarding breastfeeding, as amitriptyline is detectable in breastmilk, and in view of the potential risk for serious adverse reactions in infants, a decision should be made whether to discontinue nursing or discontinue the medicine.

### 4.2. Nortriptyline

#### 4.2.1. Studies Focused on the Treatment of Neuropathic Pain

We identified a study that reported a sex-gender difference in the intensity of neuropathic pain (396 women; 297 men) [37]. However, this difference was not modified by nortriptyline [37].

#### 4.2.2. Studies Focused on Diseases Other than Neuropathic Pain

We identified an RCT aimed at investigating potential sex-gender differences in the side effects induced by nortriptyline in 78 outpatients (45 women; 33 men) with major depression [38]. The results showed that nortriptyline treatment induced a greater increase in heart rate in men than in women, while a higher rate of women than men reported experiencing a dry mouth (see Table 1) [38]. The authors reported that, after the first dose, women achieved higher nortriptyline levels than men; then, the daily doses were adjusted to maintain therapeutic plasma levels without sex-gender differences [38]. Unfortunately, the doses administered to the female and male patients to maintain similar plasma levels were not reported [38].

Another study investigated the association between body weight, gender, and response to antidepressants, including nortriptyline [39]. This study (212 women; 118 men) evaluated the heights and weights of patients with major depression who received nortriptyline for twelve weeks. The results showed that body mass index and obesity predicted a poor response to nortriptyline in both men and women. However, this study lacked a placebo-controlled group [39].

#### 4.2.3. Data Sheet

The nortriptyline data sheet only provides specific recommendations for women during pregnancy and lactation (see Table 1). Although stating that the safety of using nortriptyline during pregnancy and lactation has not been established, nortriptyline is one of the preferred antidepressants for use during breastfeeding [40].

### 4.3. Duloxetine

#### 4.3.1. Studies Focused on the Treatment of Neuropathic Pain

One descriptive review investigated the pharmacokinetics of duloxetine and its potential interactions with other medications [41]. This study reviewed different trials in which participants received duloxetine for the treatment of several disorders, including diabetic neuropathic pain, concluding that its pharmacokinetics can be influenced by sex, among other factors [41]. One of the studies reviewed was a pharmacokinetic meta-analysis that included 274 blood samples from 43 women and 69 men who received the same dose of duloxetine (60 mg) for the treatment of neuropathic pain [42]. Analysis revealed that women achieved higher concentrations of duloxetine compared to men (see Table 1). This finding could be due to the lower activity of the enzyme CYP1A2, involved in duloxetine metabolism [43], in women compared to that in men, suggesting that, when using similar doses, women may respond better to duloxetine than men. Nevertheless, pooling data from five different RCTs, another study found no differences between men and women in the efficacy of duloxetine in reducing pain intensity [44]. Among these five studies, three RCTs specifically evaluated the efficacy of duloxetine in the treatment of neuropathic pain, administering the same doses to 492 women and 647 men (see Table 1) [45,46,47]. Unfortunately, these studies failed to provide the breakdown of their results according to gender.

#### 4.3.2. Studies Focused on Diseases Other than Neuropathic Pain

A recent SR (29 RCTs; 2043 patients, of which more than 1100 were women) investigated the efficacy of duloxetine in the treatment of postoperative acute pain without reporting sex-gender differences [48]. The results showed a significantly lower level of postoperative pain in people receiving duloxetine compared to those receiving a placebo, even among female patients alone [48]. The authors concluded that these results are not generalizable to all patients according to a series of factors (e.g., the type of surgery) or gender [48]. Two other narrative reviews focused on the treatment of fibromyalgia confirmed the efficacy and safety of duloxetine, particularly for women [49,50].

#### 4.3.3. Data Sheet

The duloxetine data sheet provides information for women that is not limited to information regarding pregnancy and lactation (see Table 1). Regarding pregnancy, duloxetine should only be used if the potential benefit justifies the potential risk to the fetus; use is not recommended during lactation as its safety in infants is not known. Furthermore, the data sheet reports that due to it having a similar half-life in men and women, no dosage adjustment of duloxetine is required based on gender.

Duloxetine use is considered safe during breastfeeding according to the low serum levels found in breastfed infants [51]. Accordingly, the use of duloxetine should not be considered a reason to discontinue breastfeeding, but the infant should be monitored.

### 4.4. Venlafaxine

#### 4.4.1. Studies Focused on the Treatment of Neuropathic Pain

We found no studies investigating or reporting evidence of sex-gender differences in the efficacy and safety of venlafaxine to treat neuropathic pain.

#### 4.4.2. Studies Focused on Diseases Other than Neuropathic Pain

One descriptive review focused on post-traumatic stress disorder (PTSD) reported that sex and gender apparently do not influence venlafaxine’s efficacy in the treatment of this disorder [52]. This review analyzed two studies (see Table 1): in the first RCT, 178 women and 151 men received flexible doses of venlafaxine (37.5–300 mg/d) or a placebo for 24 weeks [53]; in the second one, 538 patients (65–70% of all recruited patients were females; the numbers of men and women are not provided) received a placebo or flexible doses of venlafaxine ER (37.5–300 mg/d) or sertraline for 12 weeks or less [54]. Unfortunately, neither study provided the doses administered to women and men, or divided the results according to gender [53,54]. On the other hand, one retrospective study found sex-gender differences in venlafaxine’s pharmacokinetics [55]. Using a database of the blood concentrations of patients (450 women; 287 men) who had received venlafaxine, the study found that plasma concentrations were higher in women compared to those in men (see Table 1) [56]. Similar data had previously been reported in a retrospective sample (304 women; 174 men) [57]. In agreement with this finding, an analysis of the blood samples of patients (865 women; 552 men) taking venlafaxine for depression revealed that women required a 24% lower dose compared to men to achieve similar plasma concentrations [58].

#### 4.4.3. Data Sheet

The venlafaxine data sheet affirms that it should only be used if the potential benefit justifies the potential teratogenic risk (see Table 1). It also reports that venlafaxine may pass into breastmilk. However, the sheet does not recommend dose adjustments for men and women.

### 4.5. Gabapentin

#### 4.5.1. Studies Focused on the Treatment of Neuropathic Pain

One study observed sex-gender differences in response to gabapentin used to treat postherpetic neuralgia [59]. This study included data from 335 women and 221 men, with men being more likely to report a reduction in pain intensity than women [59]. Nevertheless, the authors concluded that sex-gender was not a significant influencer of clinical response to gabapentin, since the pain intensity at baseline was the main influencing factor regardless of sex [59]. By analyzing the same sample of patients, other authors observed a sex-gender difference in gabapentin’s safety [60]. Women were significantly more likely to report adverse events compared to men, with female sex being the only significant predictor of adverse events [60].

#### 4.5.2. Studies Focused on Diseases Other than Neuropathic Pain

One study conducted with healthy subjects (18 women, 18 men; Table 1) found no sex-gender differences in gabapentin’s pharmacokinetics following the administration of the same single oral dose [61]. A larger study (121 women; 68 men) confirmed the lack of sex-gender differences in the concentration–dose ratio of gabapentin used, at similar doses, to treat epilepsy (see Table 1) [62].

#### 4.5.3. Data Sheet

The gabapentin data sheet recommends that women planning a pregnancy inform their physician and receive pre-pregnancy counseling, due to preclinical studies revealing a potential risk for the fetus. As such, gabapentin should only be used if the benefit for the mother clearly outweighs the risk for the fetus (see Table 1). Moreover, gabapentin can pass into breastmilk, although no clear effects on nursing infants have been displayed [63].

### 4.6. Pregabalin

#### 4.6.1. Studies Focused on the Treatment of Neuropathic Pain

One Japanese study analyzed a large database of patients (23,246 women; 22,085 men) who received pregabalin for the first time for the treatment of neuropathic pain (17,166 patients) and other pain conditions [64]. The results showed that women required lower doses of pregabalin than men [64]. Another complex study combined data deriving from RCTs and observational studies to predict the response to pregabalin in the treatment of neuropathic pain [65]. The results revealed sex-gender differences in a specific category of patients (cluster 5; 75 women and 162 men) but not in the other clusters [65]. The main difference between patients of these clusters was that all participants in cluster 5 were also taking another medication (gabapentin), while no patients in the other clusters were taking other medications to treat neuropathic pain. Unfortunately, the study did not provide the doses administered to men and women and the direction of the difference [65].

#### 4.6.2. Studies Focused on Diseases Other than Neuropathic Pain

Analyzing the blood samples of 94 female and 73 male epileptic patients receiving similar doses of pregabalin, one study found higher blood levels of pregabalin in women than men (the concentration–dose ratio was 42% higher in women than men; see Table 1) [62]. This result was confirmed by a pooling of four studies (2568 women and 191 men) evaluating the efficacy of pregabalin on fibromyalgia (see Table 1) [66]. This study estimated that men achieve 50.4% lower pregabalin concentrations than women [66].

#### 4.6.3. Data Sheet

The pregabalin data sheet shows the potential risk of fetal harm, and women should be informed accordingly (see Table 1). One pharmacokinetic study found that pregabalin in breastmilk recovered over a 24 h period [67]. Nonetheless, given the potential risk of tumorigenicity derived from preclinical studies, breastfeeding is not recommended during treatment with pregabalin [67].

### 4.7. Capsaicin

#### 4.7.1. Studies Focused on the Treatment of Neuropathic Pain

We did not find studies investigating or reporting evidence of sex-gender differences in the efficacy and safety of capsaicin to treat neuropathic pain.

#### 4.7.2. Studies Focused on Diseases Other than Neuropathic Pain

One experimental study using intradermal injections of capsaicin to induce trigeminal sensitization found sex-gender differences in pain intensity and distribution [68]. This study (14 women; 14 men) observed that the capsaicin-induced area of dynamic mechanical allodynia was larger in women than in men and that, among women, this area was larger during the menstrual phase compared to during the luteal phase, indicating the relevance of sexual hormones [68]. Another study confirmed that women perceive higher-intensity pain than men even after topical capsaicin [69]. A different study evaluated the role of genetic functional variants of the enzyme catechol-O-methyltransferase (COMT, involved in the metabolism of numerous molecules, including epinephrine, norepinephrine, and dopamine) in mediating sex-gender differences in capsaicin-induced pain [70]. The results showed that a specific COMT variant increased capsaicin-induced pain perception in women, but not in men [70]. Other studies observed sex-gender differences in capsaicin-induced pain (164 women and 41 men) [71], the prevention of cardiovascular diseases (13 women and 25 men) [72], and chronic itchiness [34].

#### 4.7.3. Data Sheet

Regarding the use of topical capsaicin during pregnancy or breastfeeding, the data sheet indicates a lack of high-quality data on its safety, recommending a cautious prescription (see Table 1). Nonetheless, topical use by the mother is not expected to expose either the fetus or breastfed infant to the compound; therefore, capsaicin is not contraindicated.

### 4.8. Lidocaine

#### 4.8.1. Studies Focused on the Treatment of Neuropathic Pain

No studies investigating or reporting evidence of sex-gender differences in the efficacy and safety of lidocaine to treat neuropathic pain were found.

#### 4.8.2. Studies Focused on Diseases Other than Neuropathic Pain

One observational study (32 women; 20 men) found higher lidocaine serum levels in men than in women after topical application of lidocaine patches (see Table 1) [73]. In agreement with this potential sex-gender difference, another study (10 female and 10 male subjects, all healthy) found that intramuscular lidocaine infusion reduced the pain intensity induced by hypertonic saline injection in men but not in women [74]. Conversely, a larger survey (318 women; 816 men) found that women were more likely to respond to inhaled lidocaine than men in the treatment of cluster headaches [75]. Another study (69 women and 20 men; see Table 1) observed that women develop adverse events more frequently than men (87% of the adverse events induced by lidocaine injection to treat occipital neuralgia were observed in women) [76]. Finally, two studies found no sex-gender differences related to the use of lidocaine. The first one did not detect sex-gender differences in the plasma concentrations of lidocaine among 18 patients (6 premenopausal women; 6 postmenopausal women; 6 men) who received intravenous lidocaine (1 mg/kg) before general anesthesia [77]; the second one did not report sex-gender differences in the analgesic efficacy of lidocaine in a sample of 12 male and 12 female volunteers [78].

#### 4.8.3. Data Sheet

The lidocaine data sheet affirms that no adequate studies on its safety in pregnancy have been performed, and therefore it should be used carefully (see Table 1). Moreover, as it is secreted in breastmilk, caution should be exercised when administering it to a nursing mother.

### 4.9. Tramadol

#### 4.9.1. Studies Focused on the Treatment of Neuropathic Pain

Two studies reported a sex-gender difference in the vulnerability to develop tramadol-associated adverse events [55,79]. A recent Spanish study found a higher risk for women to develop tramadol-associated vomiting compared to men among patients with chronic non-cancer pain (the type of chronic pain is not specified) [55]. This study analyzed a pharmacovigilance database including 2746 people taking tramadol, unfortunately without specifying the numbers of women and men [55]. Another study conducted in the United States (2296 women; 1427 men) also observed that women develop tramadol-associated adverse reactions more frequently than men [79].

#### 4.9.2. Studies Focused on Diseases Other than Neuropathic Pain

One narrative review investigated potential sex-gender differences in the influence of cytochrome P450 2D6 (CYP2D6) phenotypes on both the efficacy and safety of some opioids, including tramadol [80]. As CYP2D6 is involved in tramadol metabolism, according to this review, CYP2D6 influences the plasma concentration of the compound without sex-gender differences [80]. On the other hand, a Chinese study conducted on healthy volunteers (25 women; 25 men) found higher plasma concentrations in women than in men after the administration of the same oral dose of tramadol (see Table 1) [81]. An observational study reported higher rates of women than men among tramadol users in Denmark, Norway, and Sweden [82]. The same study also noted that in Denmark and Norway, women received higher average daily doses per year than men, whereas no sex-gender differences in doses were observed in Sweden [82]. Another study conducted in Iran among people (9 women; 350 men) with tramadol overdoses (i.e., 500-4000 mg daily) found that, compared to women, men more frequently developed seizures related to tramadol overdose [83]. In accordance with this observation, another Iranian study—analyzing the blood samples of people (29 women; 91 men) with tramadol intoxication—observed higher concentrations of a tramadol metabolite (N-desmethyltramadol) in men than in women [84]. This sex-gender difference may account for the higher prevalence of seizures in men (see Table 1) [83,84]. However, another study found no sex-gender differences in tramadol clearance among 295 blood samples [85], but unfortunately, this study did not provide the numbers of women and men studied. Other small clinical trials ([86]: 10 women and 10 men; [87]: 12 women and 12 men) have affirmed that tramadol and its metabolites have similar pharmacokinetics in both sexes.

#### 4.9.3. Data Sheet

The tramadol data sheet specifies that the medication should be used in pregnancy only if the possible benefits outweigh the risk for the fetus (see Table 1). It is not recommended when breastfeeding since its effects on children have not been studied. In addition, it is found in breastmilk after intravenous administration. The data sheet also advises caution when administering tramadol concomitantly with CYP2D6 and/or CYP3A4 inhibitors.

### 4.10. Tapentadol

#### 4.10.1. Studies Focused on the Treatment of Neuropathic Pain

Small sex-gender differences in the efficacy of extended-release tapentadol in the treatment of diabetic neuropathic pain have been described [88]. In detail, pooling two similar RCTs including 141 women and 202 men, a higher decrease in pain intensity was obtained in women than in men; nonetheless, this difference was not deemed clinically significant [88].

#### 4.10.2. Studies Focused on Diseases Other than Neuropathic Pain

An analysis of more than 11,000 blood samples of 1339 women and 488 men with acute pain showed a higher oral clearance of tapentadol in women than in men (see Table 1) [89]. However, this study found that only hepatic function may be considered a clinically relevant influencing factor [89]. Another retrospective cohort study found no sex-gender differences in the gut transit of oral tapentadol in patients (66 women; 34 men) undergoing laparoscopic sleeve gastrectomy [90].

#### 4.10.3. Data Sheet

Given the potential risk of severe adverse events, the use of tapentadol in pregnancy is recommended only when the potential benefit justifies the potential risk to the fetus (see Table 1). Tapentadol can be detected in breastmilk, although limited data are available on this. Four cases of breastfed infants exposed to tapentadol have been reported without untoward effects.

## 5. Discussion

To our knowledge, this is the first descriptive review aimed at evaluating potential sex-gender differences in the responses of patients to medications recommended for the treatment of neuropathic pain. As we hypothesized, sex-gender differences in the efficacy and safety of medications recommended for the treatment of neuropathic pain have been described. However, most of these differences have been identified in studies focused on the treatment of diseases other than neuropathic pain.

The most surprising finding was the observation that very few articles have specifically investigated the influence of sex and gender on responses to the pharmacological treatment of neuropathic pain. In other words, despite the very high prevalence of neuropathic pain [4] and its higher frequency among women compared to men [8], the potential influence of sex and gender on patients’ responses to the medications recommended for treating this pain condition has been neglected. Indeed, sex-gender differences have also been described in the pharmacokinetics, pharmacodynamics, efficacy, and safety of multiple medications [14]. In addition, the response to medications is purportedly dependent on hormonal status [91].

The results of the present review confirm the presence of sex-gender differences for some of these medications, being mainly observed when used to treat other clinical situations such as depression or acute pain. In detail, our review found sex-gender differences in the pharmacokinetics of amitriptyline, duloxetine, venlafaxine, gabapentin, pregabalin, and tapentadol. Globally, women seem to achieve higher blood concentrations than men during treatment with amitriptyline, nortriptyline, duloxetine, venlafaxine, and pregabalin. Sex-gender differences are found in the safety profiles of gabapentin, lidocaine, and tramadol, with a higher susceptibility in women compared to men. Globally, the sex-gender differences identified here would suggest initially administering smaller doses of these medications to women with neuropathic pain compared to those administered to men as, after the administration of similar doses to both men and women, women may achieve higher blood levels or a higher number of side effects compared to men.

Regarding the safety of these medications during pregnancy and breastfeeding, information from data sheets suggests that their use should be avoided. Overall, little or no evidence has been produced on the safe use of these compounds in pregnancy, and, globally, they should only be used when the potential benefit for the mother surpasses the potential risk for the fetus. The majority of the studied compounds are detectable in breastmilk, with several (duloxetine, pregabalin, tramadol, and tapentadol) being contraindicated for use in breastfeeding women. Further, some of these medications (amitriptyline and venlafaxine) may induce serious adverse events in infants, whilst for others (capsaicin, gabapentin, and nortriptyline), insufficient evidence has been produced in breastfeeding women.

The management of pharmacological strategies for the treatment of chronic neuropathic pain encompasses a pharmacotherapeutic armamentarium in addition to adjunctive measures, including physical therapy and, in select cases, surgical interventions [2,16,17,18]. The intricate interplay of somatosensory and affective dimensions underscores the imperative for a multidimensional therapeutic approach, cognizant of the intricate neurobiological underpinnings, to efficaciously address the complexities of neuropathic pain and ameliorate its profound impact on the individual’s holistic well-being [92,93]. Several studies have described a higher prevalence of and a higher sensitivity to neuropathic pain among women than men [6,7]. The neuroimmune basis for neuropathic pain has also been described as sexually dimorphic [11]. As an example, sex-gender differences have been described in both microglia cells and macrophages, the main immune cells involved in the pathogenesis of neuropathic pain. Interestingly, some subpopulations of these cells suppress rather than promote injury-induced pain hypersensitivity [11]. Despite this large body of evidence underlining the influence of sex and gender in this frequent and severe pain condition, the results of the present review confirm an overwhelming neglect of this factor. Globally, the present findings underline the impellent need for future studies focused on the treatment of neuropathic pain and other typologies of chronic pain to recruit appropriate numbers of women and men, particularly when considering the increased frequency of pain in women. Indeed, still today, the majority of studies undertaken to investigate pain mechanisms and treatments are performed almost exclusively on men and male animals [94,95]. However, science needs to go beyond providing for the mere inclusion of women and female animals and ensure that all phases of preclinical and clinical research are projected and performed wearing gender glasses, as suggested by the international literature [96,97].

## 6. Conclusions

The results of this review reveal the extreme paucity of studies specifically focused on sex-gender differences in the evaluation of the efficacy and safety of medications recommended for the treatment of neuropathic pain. As most patients affected by neuropathic pain fail to achieve an adequate response to the medications recommended for this condition, knowing whether these medications present sex-gender differences in their efficacy and safety would allow physicians to select the most suitable medications for both men and women in terms of both efficacy and safety.

## 7. Future Directions

There is an urgent need to conduct specific clinical trials to evaluate potential sex-gender differences among medications used to treat neuropathic pain, with the aim of improving the provision of care to patients affected by debilitating pain.

## Figures and Tables

**Table 1:** Sex-gender differences of medications recommended for treating chronic neuropathic pain.

	NNT	Sex and Gender Differences Found			Pregnancy (Risk Category)	Breastfeeding	Suggestions
Medication		Pharmacokinetic	Efficacy	Adverse Events			
First-Line Treatments							
Amitriptyline	3.6 (combined tricyclic antidepressants)	YES Blood levels: F > M (396 F; 297 M)	UNCLEAR No information found	UNCLEAR Sample of participants too small	C	Detectable in breastmilk. Potential serious adverse reactions in infants.	Doses required: F < M
Nortriptyline		YES Blood levels: F > M (45 F; 33 M)	UNCLEAR No information found	UNCLEAR Different adverse events: F ≠ M	C	Low levels in breastmilk. Use is possible with caution.	Doses required: F < M
Duloxetine	6.4 (combined serotonin–noradrenaline reuptake inhibitors)	YES Blood levels: F > M (43 F; 69 M)	NO Pain reduction: F = M (492 F; 647 M)	UNCLEAR No information found	C	Not recommended.	Doses required: F < M
Venlafaxine		YES Blood levels: F > M (450 F; 287 M; and 304 F; 174 M) Dose required to achieve similar blood levels: F < M (865 F; 552 M)	NO Pain reduction: F = M (178 F; 151 M)	UNCLEAR No information found	C	Detectable in breastmilk. Potential serious adverse reactions in infants.	Doses required: F < M
Gabapentin	7.2	NO Blood levels: F = M (18 F; 18 M; and 121 F; 68 M)	UNCLEAR A difference found related to baseline pain intensity	YES F > M (335 F; 221 M)	C	Detectable in breastmilk. Unknown adverse reactions in infants.	Doses required: F < M
Pregabalin	7.7	YES Blood levels: F > M (94 F; 73 M; and 2568 F; 191 M) Dose required: F < M (23,246 F; 22,085 M)	UNCLEAR A difference found without information on the direction	UNCLEAR No information found	C	Low levels in breastmilk. Not recommended (risk of tumorigenicity).	Doses required: F < M
Second-Line Treatments							
Capsaicin	10.6	UNCLEAR No information found	UNCLEAR No information found	UNCLEAR No information found	B–C	Not studied.	Further studies needed
Lidocaine	Not reported	UNCLEAR Blood levels: F < M (32 F; 20 M; topical); F = M (12 F; 6 M; intravenous)	UNCLEAR Pain reduction: F < M (10 F; 10 M); F > M (318 F; 816 M); F = M (12 F; 12 M)	YES F > M (69 F; 20 M; injected for occipital neuralgia)	B	Detectable in breastmilk. Use is possible with caution.	Doses required: F < M
Tramadol	4.7	UNCLEAR Blood levels: F > M (25 F; 25 M); F = M (10 F; 10 M; 12 F; 12 M); F < M (9 F; 350 M; and 29 F; 91 M) Dose administered: F > M (number of F and M unknown); F = M (number of F and M unknown)	UNCLEAR No information found	YES F > M (2746 people, especially vomiting; 2296 F; 1427 M)	C	Detectable in breastmilk (after intravenous administration). Not recommended (insufficiently studied).	Doses required: F < M
Third-Line Treatments							
Tapentadol	10.2	UNCLEAR Oral clearance: F > M ^1^ (1339 F; 488 M) Gut transit: F = M (66 F; 34 M)	UNCLEAR Pain reduction: F > M (141 F; 202 M; non-significant difference)	UNCLEAR No information found	C	Detectable in breastmilk. Not recommended (insufficiently studied).	Further studies needed

**Legend:** F: females; M: males; NNT: number of patients needed to be treated to obtain therapeutic effect in one patient (lower NNT means better efficacy). Pregnancy Risk Categories: B: No risk in animal studies (there are no adequate studies in humans, but animal studies have not demonstrated a risk to the fetus); C: Risk cannot be ruled out (there are no satisfactory studies in pregnant women, but animal studies have demonstrated a risk to the fetus; potential benefits of the drug may outweigh the risks). ^1^ According to [18], sex-gender is non-independently associated.

## Data Availability

Data sharing is not applicable to this article as no datasets were generated or analyzed during the current study.
